# Editorial: Moyamoya disease

**DOI:** 10.3389/fneur.2022.1071610

**Published:** 2022-11-30

**Authors:** Yuxiang Gu

**Affiliations:** Huashan Hospital, Fudan University, Shanghai, China

**Keywords:** moyamoya disease (MMD), cerebral vascular disease, stroke, EC-IC bypass, hemodynamics

Moyamoya disease is a rare cause of stroke characterized by progressive stenosis of the terminal carotid arteries and compensatory collaterals. In the last decade, new techniques in clinical contrast-enhanced imaging have allowed for a more accurate and useful diagnosis using CT, MRI, and DSA images. However, Moyamoya disease remains to this day an unknown etiology, mainly because diagnosis is based on the exclusion of all other possible underlying medical conditions.

This Research Topic covers some recent investigations in research on Moyamoya disease, covers original research, and reviews at the physiological, etiology, and surgical treatment levels. It contains 10 articles, including 3 reviews.

Mineharu and Miyamoto present a review focused on RNF213/Mysterin and GUCY1A3 and their strong linker, calcineurin/NFAT signaling, and caveolin to understand the pathophysiology of moyamoya disease. Although intimal thickening with fibrosis and damaged vascular smooth muscle cells are the distinguishing features of moyamoya disease, the origin of the fibrous tissue and the mechanism of smooth muscle cell damage remains not fully elucidated. The review also points out that endothelial cells and smooth muscle cells have long been a focus of interest, but other vascular components such as immune cells and the extracellular matrix also need to be investigated in future studies.

Moyamoya disease is a complex and incompletely-understood cerebrovascular pathological entity that requires thorough clinical and imaging evaluation. Larson et al. described their institution's implementation of, rationale for, and experience with a comprehensive multidisciplinary collaboration and evaluation strategy for adult patients with moyamoya.

A major difficulty in treating moyamoya is the lack of effective methods to detect novel or progressive disease prior to the onset of disabling stroke. More importantly, a tool to better stratify operative candidates and quantify response to therapy could substantively complement existing methods. Sesen et al. present proof-of-principle data supporting the use of urinary biomarkers as diagnostic adjuncts in pediatric moyamoya patients. The authors summarized that urinary proteins are useful predictors of the presence of moyamoya and may provide a basis for a novel, non-invasive method to identify new disease and monitor known patients following treatment.

Fox et al. present a review of the pathological features of the stenosis associated with MMD. Neointimal hyperplasia, disruption of the internal elastic lamina, and medial attenuation, which ultimately lead to progressive decreases in both luminal and external arterial diameter. The authors summarize the molecular pathways which have been implicated in the pathophysiology of stenosis in MMD with functions in cellular proliferation and migration, extracellular matrix remodeling, apoptosis, and vascular inflammation. The author also raised several questions for further investigation.

In the study by Hurth et al., the clinical value of early postoperative computed tomographic angiography (CTA) after direct extracranial-intracranial (EC-IC) bypass surgery in moyamoya patients was investigated. The authors summarized that early postoperative CTA has a high predictive value to confirm the patency of a bypass. On the other hand, a high false positive rate of (according to CTA) occluded bypasses after direct EC-IC bypass surgery can be seen.

Lucia et al. presented research aimed at characterizing the cases of bypass failure and repeat revascularization at a single center. A rescue surgery should be considered in those with neurological symptoms and decreased CVRC. Intermediate flow bypass using a radial artery graft is a reliable technique for patients requiring repeat revascularization.

In the study by Chen et al., A bibliometric analysis to examine the development of and research trends in MMA research was carried out. The research trends of global scientific research on MMA over the past decade were systematically analyzed. The study can provide guidance for scholars who want to understand current trends in research in this area and new research frontiers.

Inflammation has been shown to play a pivotal role in the pathogenesis of moyamoya disease. Ma et al. investigated the relationship between Platelet-to-lymphocyte ratio (PLR) and neutrophil-to-lymphocyte ratio (NLR) in Chinese patients with newly diagnosed MMD. In patients with MMD, there seemed to be a positive association between PLR and NLR. This may help to further explain the role of inflammation in the occurrence and development of MMD.

In the study by Zheng et al., the hemodynamic changes using ultrasound according to digital subtraction angiography (DSA) findings and the association between ultrasound parameters and clinical symptoms of moyamoya disease (MMD) were investigated. The authors evaluated Hemodynamic parameters of the extracranial internal carotid artery (EICA) and posterior cerebral artery (PCA) by ultrasound, and they found that Ultrasound parameters were related to DSA findings, ultrasound may be useful in predicting the clinical symptoms of patients with MMD.

Ye et al. presented a study aimed to investigate the effectiveness and safety of antiplatelet therapy compared with conservative treatment and surgical revascularization in ischemic MMD patients. Antiplatelet agents were effective and safe in preventing further cerebral ischemic attacks in adult patients with ischemic MMD. They may be a replacement therapy for patients with surgical contraindications and patients prior to revascularization.

## Author contributions

The author confirms being the sole contributor of this work and has approved it for publication.

## Conflict of interest

The author declares that the research was conducted in the absence of any commercial or financial relationships that could be construed as a potential conflict of interest.

## Publisher's note

All claims expressed in this article are solely those of the authors and do not necessarily represent those of their affiliated organizations, or those of the publisher, the editors and the reviewers. Any product that may be evaluated in this article, or claim that may be made by its manufacturer, is not guaranteed or endorsed by the publisher.

